# Exploring the Mechanism of Flavonoids Through Systematic Bioinformatics Analysis

**DOI:** 10.3389/fphar.2018.00918

**Published:** 2018-08-15

**Authors:** Tianyi Qiu, Dingfeng Wu, LinLin Yang, Hao Ye, Qiming Wang, Zhiwei Cao, Kailin Tang

**Affiliations:** ^1^Institute of Biomedical Sciences, Fudan University, Shanghai, China; ^2^School of Life Sciences and Technology, Tongji University, Shanghai, China; ^3^Hebei Key Laboratory of Metabolic Diseases and Clinical Medicine Research Center, Hebei General Hospital, Hebei, China; ^4^Sinotech Genomics Ltd., Shanghai, China; ^5^East China University of Science and Technology, Shanghai, China

**Keywords:** flavonoids, mechanism of action, pathway analysis, protein–protein interaction network, structure activity relationship

## Abstract

Flavonoids are the largest class of plant polyphenols, with common structure of diphenylpropanes, consisting of two aromatic rings linked through three carbons and are abundant in both daily diets and medicinal plants. Fueled by the recognition of consuming flavonoids to get better health, researchers became interested in deciphering how flavonoids alter the functions of human body. Here, systematic studies were performed on 679 flavonoid compounds and 481 corresponding targets through bioinformatics analysis. Multiple human diseases related pathways including cancers, neuro-disease, diabetes, and infectious diseases were significantly regulated by flavonoids. Specific functions of each flavonoid subclass were further analyzed in both target and pathway level. Flavones and isoflavones were significantly enriched in multi-cancer related pathways, flavan-3-ols were found focusing on cellular processing and lymphocyte regulation, flavones preferred to act on cardiovascular related activities and isoflavones were closely related with cell multisystem disorders. Relationship between chemical constitution fragment and biological effects indicated that different side chain could significantly affect the biological functions of flavonoids subclasses. Results will highlight the common and preference functions of flavonoids and their subclasses, which concerning their pharmacological and biological properties.

## Introduction

Flavonoids are a family of phenolic substances sharing the same backbone structure of 2-pheny1-1,4-benzopyronemay, which are very abundant in nature, being accumulated in regular human diets including flowers (Zhang and Ma, 2018), fruits (Chang et al., 2018), vegetables, tea, wine (Matveeva et al., 2018), and so on (Szmitko and Verma, 2005). With the basic core scaffold, flavonoids have been demonstrated to exhibit relevant biological properties involving strong activity for anti-oxidant (Pietta, 2000), anti-allergy (Kawai et al., 2007; Castell et al., 2014), anti-inflammatory (Nijveldt et al., 2001; Serafini et al., 2010; Matias et al., 2014), anti-microbial (Cushnie and Lamb, 2005), and anti-obesity (Hughes et al., 2008) effects. Also, flavonoids have been reported to have effect on reducing the risk of cardiovascular disease (Hooper et al., 2008; Mulvihill and Huff, 2010; Feliciano et al., 2015) and cancers (Yao et al., 2011; Batra and Sharma, 2013), ameliorating cognition (Spencer et al., 2009; Williams and Spencer, 2012) and neuro-protection in Alzheimer’s disease (Bakhtiari et al., 2017; Mohebali et al., 2018). Moreover, it is also found that flavonoids act as agonist or antagonist depending on the estrogen concentrations to regulate estrogenic-like activity (Breinholt et al., 1999; Hwang et al., 2006).

On the basis of common core scaffold, various combinations of substituent chemical groups on different positions may lead to structure diversity of flavonoids. This diversity can be further increased with possible variations of different functional groups, such as hydroxyl, methoxyl, carbonyl, and olefinic groups (Gontijo et al., 2017). According to the structure variations, flavonoids can be generally assigned into six main subclasses: flavones, flavonols, flavanones, flavanols, flavan-3-ols, and isoflavones (Ross and Kasum, 2002), for which the chemical properties depend on their structural classes, degrees of hydroxylation, substitutions, conjugation, and degree of polymerization (Kumar and Pandey, 2013). However, the functional similarities and differences, as well as the structure basis of different functions for flavonoids subclasses are not fully revealed yet.

In this study, a comprehensive bioinformatics analysis was performed based on a large-scale dataset including 679 flavonoids and 481 corresponding targets to decipher the mechanism of action (MOA) of flavonoids with a new perspective. Results illustrated the structure activity relationship of different flavonoids subclasses, which hint the protective roles of flavonoids subclasses in different human diseases. With the accumulation of flavonoids and corresponding targets, it is possible to comprehensively investigate the MOA of flavonoids in a systematic level and interpret the therapeutic mechanism to guide the drug discovery from natural flavonoid products.

## Materials and Methods

### Dataset

#### Flavonoids and Corresponding Targets

**FIGURE 1:**
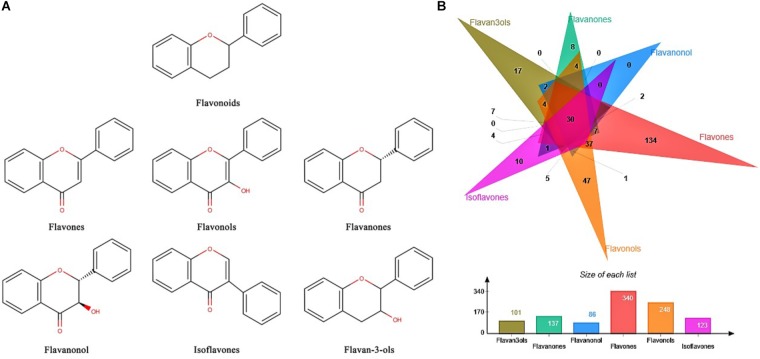
Structures and targets information of flavonoids. **(A)** Core scaffold structures of six flavonoid subclasses. **(B)** Target number of different flavonoid subclasses.

A total number of 5,006 chemical structures of natural plant products were derived from Natural Product Activity and Species Source Database (NPASS) (Zeng et al., 2018). Among them, main types of flavonoids including flavones, flavonols, flavanones, flavanonol, isoflavones, and flavan-3-ols were categorized according to the scaffold structures derived by cheminformatics software-RDKit (Landrum, 2010), which were illustrated in **Figure 1A**. Further, corresponding direct targets of flavonoids were selected from 5,337 targets of natural plant products in NPASS. After that, 679 flavonoids and 481 corresponding targets were selected and listed in **Supplementary Table 1**. Number of targets for different flavonoid subclasses were illustrated in **Figure 1B**.

**Table 1:** Main functions of six major modules.

Modules	Main function
1	Cardiovascular regulations; cell cycle regulation
2	Immune and inflammation response
3	Cell response to stimulation and hormone regulation
4	Neuromodulation and signal transduction
5	ancer and viral-related diseases
6	Process of glyoxylic acid metabolism


### Enrichment Analysis of Flavonoids’ Targets

#### Diversity Analysis of Natural Flavonoid Products’ Targets

Targets of natural flavonoid products were mapped into Kyoto Encyclopedic of Genes and Genomes (KEGGs) (Kanehisa et al., 2012) and Gene Ontology (GO) (Ashburner et al., 2000) through Metascape (Tripathi et al., 2015) to analyze their enrichment pathways. Then, the enrichment pathways were generated for six flavonoid subclasses.

#### Specific Pathway Enrichment Analysis of Natural Flavonoids Products

To distinguish the specific pathway of flavonoids from other natural plant products, permutation test was implemented 1,000 times to identify the specific pathway of flavonoids’ targets by setting the 4,327 other natural plant products as background.

### Pharmacology Network Analysis

Protein–protein interaction (PPI) networks of flavonoids’ targets were generated and modularized through Metascape (Tripathi et al., 2015). Further, the bio-functional similarity and difference between networks of six subclasses were compared based on the main functional modules. Then, PPI enrichment analysis was carried out with the following databases including BioGrid (Chatr-Aryamontri et al., 2017), InWeb_IM (Li et al., 2017), and OmniPath (Turei et al., 2016). The densely connected network components was identified by Molecular Complex Detection (MCODE) algorithm (Bader and Hogue, 2003) and viewed by Cytoscape (Shannon et al., 2003).

### Structure–Activity Relationship Analysis

In order to analyze the structure–activity relationship, basic physicochemical properties including molecular mass (weight), lipid water distribution coefficient (LogP), hydrogen bond receptor (NumHAcceptors), hydrogen bond donor (NumHDonors), rotatable bond (NumRotatableBonds), topological molecular polarity surface area (TPSA) and Lipinski’s Rule of five were calculated for different natural flavonoid products through RDKit (Landrum, 2010).

Also, the core scaffold and side chains of each natural flavonoid products were derived according to their chemical structures. Since flavones, flavonols, flavanones, flavanonol, and flavan-3-ols share the same core scaffold, the structure–activity relationships of above five subclasses were analyzed. Then, according to GO (Ashburner et al., 2000), the bio-functional annotation of each structure segment can be obtained. Further, to identify the association between chemical structure of flavonoid subclasses and biological function, structure–activity relationship was further analyzed through Apriori algorithm (Agrawal and Srikant, 1994). Here, the minimum support parameter was set as 0.01 and the minimum confidence was set as 0.5 for calculation.

## Results

### Pathway Enrichment Analysis of Flavonoids’ Targets

**FIGURE 2:**
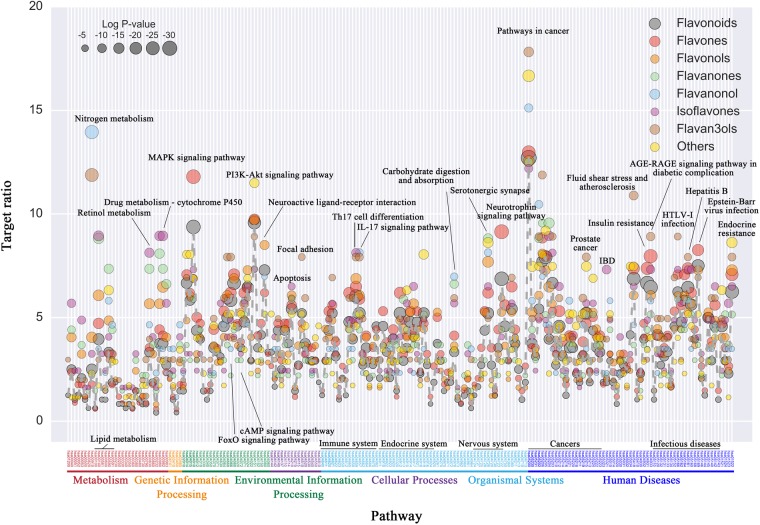
KEGG pathway enrichment analysis of natural flavonoid products’ targets. Here, *X*-axis represents the enriched pathways (*p*-value < 0.05), which were categorized according to KEGG classification. *Y*-axis represents target proportion of flavonoids in each pathway (number of flavonoids’ target in pathway/total number of flavonoids’ targets), the size of each nodes represents the significance of enrichment level (–LogP). Flavonoids and all six subclasses were marked in different colors.

The biological function of flavonoids’ target was deciphered through pathway enrichment analysis based on the background pathway dataset (**Figure 2** and **Supplementary Table 2**). Results showed that, the targets of flavonoids were enriched in multiple essential pathways including metabolism, genetic information processing, environmental information processing, cellular process, organismal systems, and multiple pathways which were related to human diseases such as infectious diseases and cancer. For instance, in environmental information processing, flavonoids were enriched in multiple cell signaling pathways including MAPK signaling pathway, PI3K-Akt signaling pathway, FoxO signaling pathway and cAMP signaling pathway. In cellular processes, flavonoids can significantly regulate pathways such as apoptosis, focal adhesion, cell cycle, and autophagy. Further, it can be found that flavonoids’ targets were significantly enriched in several organismal systems including immune system, endocrine system and nervous system. Especially for immune system related pathways, flavonoids were enriched in Th17 cell differentiations, IL-17 signaling pathway, Toll-like, and NOD-like signaling pathways. Besides, multiple flavonoids’ targets can be found in the endocrine system pathways, such as progesterone-medicated oocyte maturation, GnRH signaling, oxytocin signaling and thyroid hormone signaling pathways. Also, nervous system-related pathways such as serotonergic synapse, and neurotrophin signaling pathways were enriched by corresponding targets. Moreover, flavonoids’ targets existed in pathways of essential human diseases such as multi-cancer, insulin resistance and infectious diseases including HTLV-1 infection, Epstein–Barr virus infection and Hepatitis B.

Besides the common enrichment pathways, different flavonoid subclasses illustrated different preference. For instance, targets of flavanonol and flavan-3-ols were more significantly enriched in nitrogen metabolism pathways than other subclasses. Targets of isoflavones, flavanones, and flavonols were enriched in metabolism pathways such as lipid, retinol, and drug metabolism pathway. Flavones’ targets were significantly enriched in MAPK signaling pathway and neurotrophin signaling pathway, which means natural flavone products may have therapeutic effects on neurological-related diseases. Pervious researches indicated that flavones such as apigenin and luteolin could activate Nrf2-antioxidant response element (ARE)-mediated gene expression and induce anti-inflammatory activities through the PI3K and MAPK signaling pathways (Paredes-Gonzalez et al., 2015). Also, both compounds could significantly increase the endogenous mRNA and protein level of Nrf2 and Nrf2 targeting genes with important effects on hemo oxygenase-1 (HO-1) expression, thus, led to cytoprotective effects and neurite outgrowth (Lin et al., 2010; Zhao et al., 2013; Zhang et al., 2015). In addition, corresponding targets of flavan-3-ols and flavanonol were enriched in cancer-related pathways. Natural flavan-3-ol products such as (-)-epigallocatechin gallate (EGCG), (-)-epicatechin gallate (ECG), (-)-epigallocatechin (EGC), and (-)-epicatechin (EC) were discovered flavan-3-ols from green tea, which could provide possible prevention of cancers (Henning et al., 2013; Yang C.S. et al., 2014). Although the flavonoids contain similar biological function based on the same core scaffold, above results indicated the different biological functions of flavonoid subclasses with different chemical structures. Thus, the therapeutic selection and clinical application for flavonoid subclasses were different from each other.

### Functional Difference Between Flavonoids and Other Natural Plant Products

**FIGURE 3:**
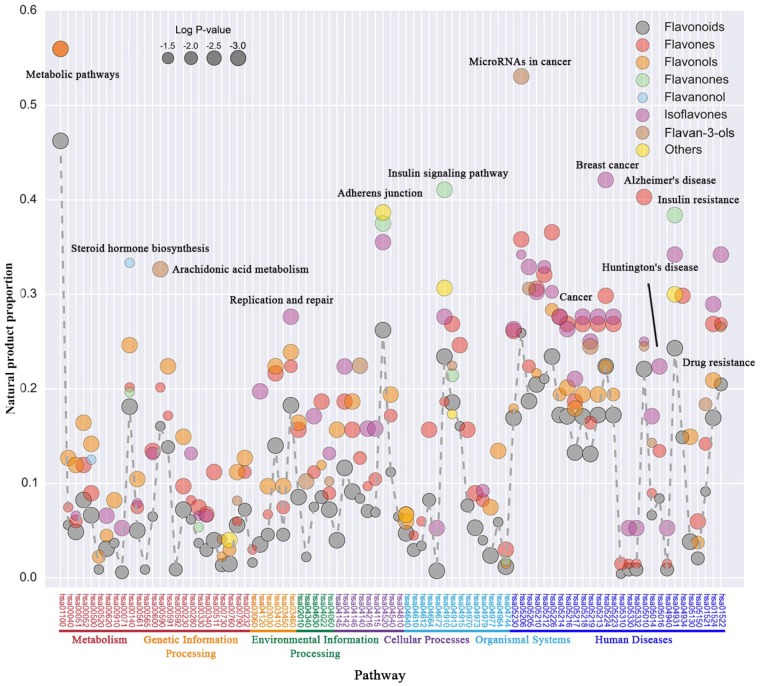
Specific KEGG pathway enrichment analysis of natural flavonoid products’ targets. Here, *X*-axis represents the enriched pathways (*p*-value < 0.05), which were categorized according to KEGG classification. *Y*-axis represents compound proportion of flavonoids in each pathway (number of target related compounds/total number of compounds in each class), the size of each nodes represented the significance of enrichment level (–LogP). Flavonoids and all six subclasses were marked in different colors.

To further discover the functional difference between flavonoids and other natural plant products, the specific enrichment pathway of flavonoids’ targets were analyzed by setting other natural plant products as background. Results showed that, flavonoids were enriched in cancer-related pathways compared with other natural products (**Figure 3** and **Supplementary Table 3**). Among them, isoflavones and flavones were enriched in multi-cancer related pathways, flavan-3-ols can regulate the pathway of microRNA in cancer and isoflavones significantly enriched in the pathway of breast cancer, indicating the potential anti-cancer preferences of flavonoid subclasses. It can be noticed that natural flavan-3-ol products such as EGCG could alter epigenetic processes through DNA methylation, histone modification and miRNA regulation such as miR-92, miR-93, miR-106, miR-7-1, miR-34a, and miR-99a (Chakrabarti et al., 2012), which could provide anti-cancer and cardiovascular protections (Henning et al., 2013; Yang C.S. et al., 2014). Also, soy isoflavones, including genistein, daidzein, and their corresponding glucosides were reported to reduce the risk of breast cancer through meta-analysis (Yamamoto et al., 2003; Dong and Qin, 2011). *In vitro*, these isoflavones could significantly restrain the growth of human breast cancer cells (Peterson and Barnes, 1991). In addition to cancer related pathways, flavonoids were enriched in metabolic, steroid hormone biosynthesis, replication and repair, adherence junction, insulin signaling and several diseases related pathways. Meanwhile, existential discrepancy was found between different flavonoid subclasses. For example, although flavonoids showed biological functions on multiple nervous system diseases, only flavones were significantly enriched in Alzheimer’ disease related pathways. Previous researches showed that the derivatives of flavone acting at different target could elicit varied pharmacological properties with various substitution patterns, including anti-oxidant, anti-cancer activity, neuroprotective activity (Singh et al., 2014). Those derivatives also showed good binding affinity to Aβ aggregates and high brain penetration, which illustrate potential therapeutic utilities for Alzheimer’s disease (Ono et al., 2005, 2007). Also, isoflavones were significantly enriched in Huntington’s diseases related pathways, which may related with isoflavones-mediated autophagy (Pierzynowska et al., 2017, 2018). Generally, both common and discrepancy were found in flavonoid enriched pathways which represent the specific biological function and potential therapeutic utility of different flavonoid subclasses.

### Network Pharmacology and Modularization Analysis

**FIGURE 4:**
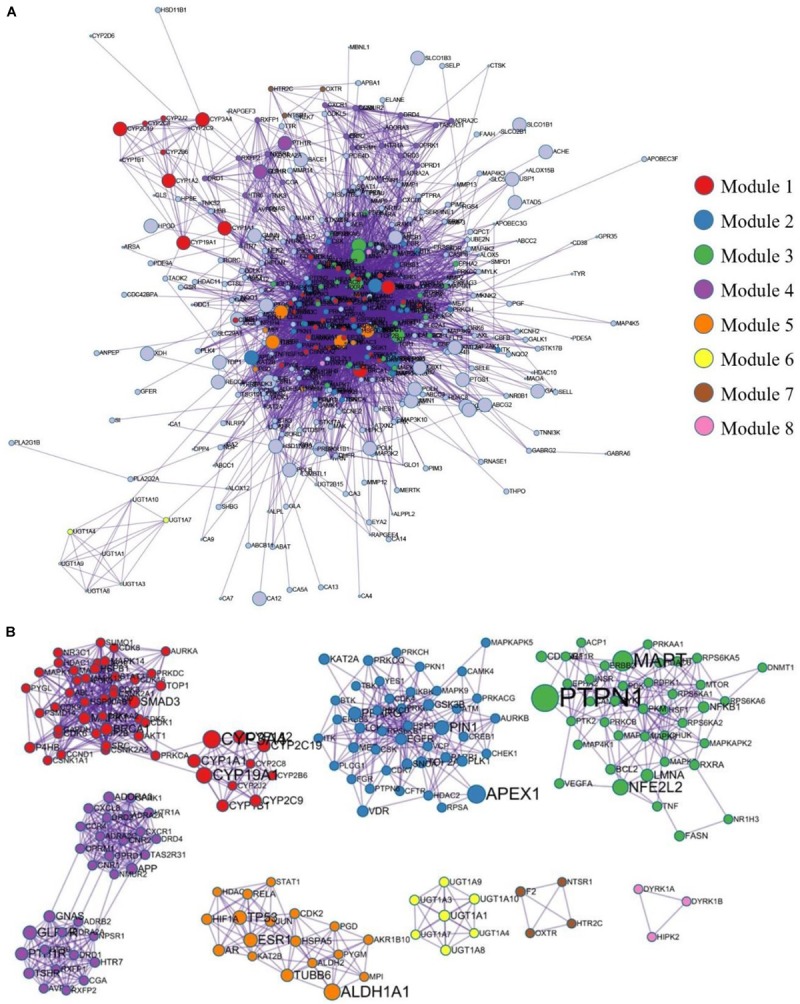
Protein–protein interaction (PPI) network of natural flavonoid products’ targets. **(A)** General PPI network of natural flavonoid products’ targets. **(B)** Modularized PPI network of natural flavonoid products’ targets. Size of each node represents the ratio (=number of target related compounds/total number of compounds) of targets. Different modules were marked in different colors.

To globally view the enrichment pathways of flavonoid, the network of all enriched targets for six flavonoids’ subclass were analyzed and decomposed into eight modules. In **Figure 4**, the size of each node represents the ratio (=number of target related compounds/total number of compounds) of targets in here. Targets mapped in the same modules were marked in the same color and network of targets in six major modules were analyzed through KEGG and GO to discover the function of flavonoid (**Table 1**), top 10 enriched pathways and GO terms were list in **Supplementary Table 4**. Entrez Gene ID and symbol in each module were listed in **Supplementary Table 5**.

Targets in module 1 were mainly enriched on epoxygenase P450 pathway, VEGF signaling pathway, fluid shear stress and atherosclerosis pathway, which related with cardiovascular regulations such as vascular dilatation. Meanwhile, the enrichment of pathways for FoxO signaling, mitotic cell cycle regulation, cell cycle arrest, negative regulation of cell cycle showed that cell cycle related functions can also been reflected in module 1. For module 2, targets were significantly enriched on pathways for T-cell receptor signaling, NF-kappa B signaling, inflammatory mediator regulation of TRP channels, immune response-regulating cell surface receptor signaling, immune response activating cell surface receptor signaling and immune response-activating signal transduction, which indicated the function of module 2 was related to immune inflammation. Further, it can be noticed that targets in module 3 were significantly enriched on pathways of insulin resistance, peptidyl-serine phosphorylation, peptidyl-serine modification, cellular response to nitrogen compound, cellular response to organonitrogen compound, positive regulation of kinase activity and cellular response to hormone stimulus, which illustrated the function of module 3 were closely related with functions of cell response to stimulation and hormone regulation. Moreover, the enrichment in neuroactive ligand-receptor interaction, cAMP signaling, calcium signaling, serotonergic synapse, cGMP-PKG signaling and dopaminergic synapse pathways reflected the targets in module 4 were related to neuromodulation and signal transduction. In addition, module 5 was found to relate with human diseases such as cancer and viral-related diseases since most targets were enriched in viral carcinogenesis pathway, cancer and infectious disease related pathways. For module 6, targets were enriched in flavonoids glucuronidation, glucuronate pathway, ascorbate and aldarate metabolism, pentose and glucuronate interconversions, which meant functions of module 6 were mainly embodied in the process of glyoxylic acid metabolism.

### Module Mapping of Different Flavonoid Subclasses

In order to understand the function differences among flavonoid subclass, targets of six flavonoid subclasses were mapped into above modules. Major nodes reflected the common targets for each flavonoid subclass.

Start from flavan-3-ols, the targets were mainly distributed in module 1, 2, 3, and 5, and generally enriched in pathways of cancer, fluid shear stress and atherosclerosis, AGE-RAGE signaling in diabetic complications, which related with cardiovascular, cell cycle regulation and cancer (**Supplementary Figure 1**). For example, MAPK 14 in module 3 was found to participate in multiple cellular processes including cell proliferation, differentiation, transcriptional regulation and development (Young, 2013). Also, it can be noted that MAPK 14 may related with atherosclerosis (Cheng et al., 2017). Further, BCL2 in module 2 was a therapeutic target for chronic lymphocytic leukemia since it can regulate lymphocyte in blood by hindering cell apoptosis (Ruefli-Brasse and Reed, 2017; Tahir et al., 2017) and PGD in module 5 was related with human cervical carcinoma (Lee et al., 2014).

Targets of flavanones mainly distributed in module 1, 4, and 5, several scattered in modules 2 and 3, which illustrated the pharmacological activities of flavanones for anti-cancer and anti-oxidant (**Supplementary Figure 2**). For example, nodes such as CYP1A1, CYP1A2, CYP1B1 in module 1 belonged to cytochrome P450 (CYPs) family, which could enrich in epoxygenase P450 pathway and relate with cardiovascular-related functions such as vascular ectasia. APEX1 in module 2 was found affecting cancer RNA metabolism and triple-negative breast cancer (Antoniali et al., 2017; Chen et al., 2017).

Targets of flavanonol were relatively less than the others and separated in different modules, which means the function of flavanonol are quite scattered (**Supplementary Figure 3**). Similar to flavanones, nodes such as CYPs in module 1 and APEX1 in module 2 were also detected in flavanonol, which indicated the potential function of it on cardiovascular and cancer related functions. Meanwhile, MAPT in module 2 was found closely related with neurodegenerative diseases, including Parkinson’s disease (Beevers et al., 2017).

Target of flavones (**Supplementary Figure 4**) and flavonols (**Supplementary Figure 5**) were distributed in all six major modules, which indicated the broad function of compounds from those two subclasses. Besides common nodes such as CYPs, APEX1, MAPT, which reflect the same function for cancer, cardiovascular and neurodegenerative as other flavonoid subclasses flavones contains other nodes such as ALDH1A1 in module 5, which reflect potential associations with cancer invasion (Yao et al., 2017; Li et al., 2018).

Specifically, isoflavones are a type of naturally occurring isoflavonoids, which act as phytoestrogens in mammals, their targets were mainly distributed in module 1, 2, and 5 (**Supplementary Figure 6**). Previous researches indicated that BRCA1 in module 1 was associated with risk of estrogen-receptor-negative breast cancer (Milne et al., 2017), NFE2L2 in module 3 was related with cell multisystem disorder (Huppke et al., 2017), and TP53 in module 5 was related with human immunodeficiency virus-related head and neck squamous cell carcinoma (Gleber-Netto et al., 2018).

### Structure Activity Relationship Analysis of Flavonoids

**FIGURE 5:**
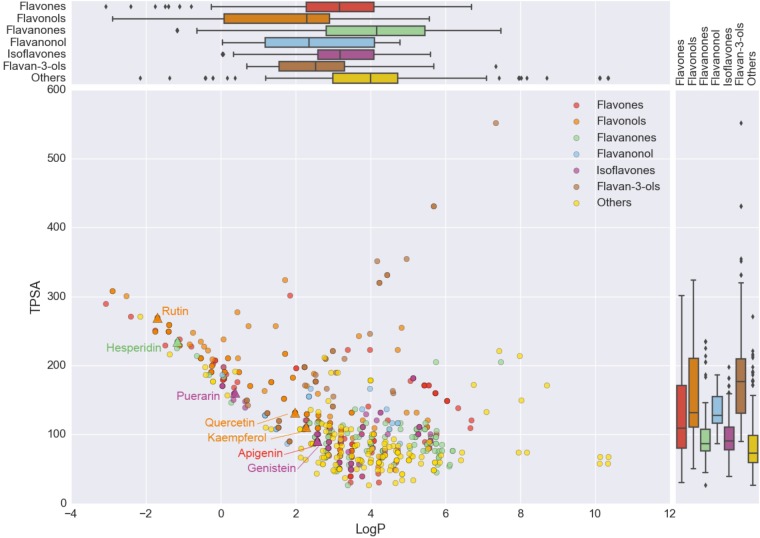
Distribution of LogP and TPSA in different flavonoids. Different color represents different flavonoid subclasses. *X*-axis represents the value of LogP, while *Y*-axis represents the value of TPSA.

In order to explore the cause of the functional similarity and difference among flavonoids’ subclasses, the structure activity relationship of different flavonoids were analyzed. By calculating the structural and physic-chemical properties of natural flavonoid products, the structure difference of flavonoids’ subclasses can be discovered to conjecture the potential effects of their biological functions (**Supplementary Figure 7**). Results showed that the rotatable bonds (NumRotatableBonds) and molecular weight (Weight) in different subclasses are quite similar, however, difference can be detected in H-bond acceptor (HAcceptor), H-bond donor (HDonor), lipid-water partition coefficient (LogP), and Topological polarity surface area (TPSA) for different flavonoid subclasses. LogP and TPSA could affect the absorption and distribution of drug, which should contain a certain degree of dissolution and appropriate lipid water distribution to be effective. Further, to provide nervous system activity, drug with larger liposolubility may be easier to pass the blood–brain barrier (BBB) (Yang Y. et al., 2014). The non-polar structural fragments such as alkyl group, halogen atom and aliphatic ring in chemical molecules will increase the liposolubility of molecules. Meanwhile, TPSA has a great impact on the cell penetration of drug molecules. In that case, the TPSA should be relatively lower for drugs which needs to across BBB and act on the receptors of central nervous system (Mehdipour and Hamidi, 2009). Natural products of flavones, flavanones, and isoflavones contain larger LogP and lower TPSA than other flavonoids, which indicated the potential activities to across the BBB (**Figure 5**). For example, apigenin of flavones, quercetin, and genistein of isoflavones, hesperidin of flavanones and rutin, quercetin, and kaempferol of flavonols would have the ability to across the BBB (**Figure 5**). Among them, genistein and apigenin could provide stronger ability to across the BBB since their larger LogP and lower TPSA (Yang Y. et al., 2014), which indicated the potential ability of other flavonoids meets the appropriate value of LogP and TPSA.

Further, in order to evaluate the drug-likeness of flavonoids, Lipinski’s Rule of Five (ROF) of different flavonoid subclasses were analyzed (**Supplementary Figure 8**). It can be found that for flavones, flavonols, flavanones, flavanonol, and isoflavones, near half of the compound can pass ROF, while for flavan-3-ols the percentage of ROF-passed compounds is extremely low, which indicated different drug-likeness of flavonoid subclasses.

**FIGURE 6:**
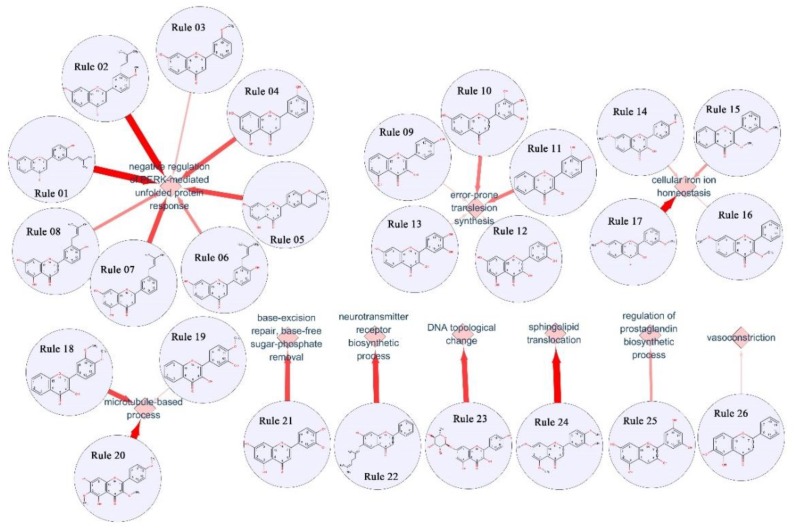
Illustration of relationship between structure patterns and functions according to structure–activity relationship analysis of flavonoids.

By excavating the relationship between chemical constitution fragment and biological effects through Apriori (Agrawal and Srikant, 1994), results showed that the core scaffold and side chain in flavonoids can significantly affect the biological functions (**Figure 6**). For example, in rule 01–07, side chain such as hydroxyl in position 1 on the core scaffold structure of flavanones may assist the negative regulation of PERK-mediated unfolded protein response. Also, in rule 09–13, hydroxyl side chain in position 1, 3, 10, 11, and 12 on the core scaffold structure of flavonols closely related with error-prone translesion synthesis. Among them, natural products such as myricetin, robinetin, tricetin could against hydrogen peroxide-induced DNA damage and might reduce the risk of multiple cancers (Huang and Ferraro, 1992; Shelby et al., 1997). Meanwhile, natural products with core scaffold of flavonols and oxygen methyl on different positions as side chain illustrated the bio-function of cellular iron ion homeostasis (rule 14–17) and microtubule-based process (rule 18–20). Besides above rule of generality, individual rules can also be found in **Figure 6**. For example, the core scaffold of flavones combined with hydroxyl side chain in position 1, 3, 11, and 12 will related with base-excision repair and base-free sugar-phosphate removal (rule 21). Previous studies indicated that the number and position of glycoside and hydroxyl groups in flavonoids would affects the ability of permeation (Yang Y. et al., 2014). We also found that hydroxyl side chain in position 1 and 3 combined with hydrocarbyl side chain in position 2 which related with neurotransmitter receptor biosynthetic process (rule 22) will increase the liposolubility and enhance its transmembrane abilities. It can be noted that, the bio-activity of molecules which meet rule 22 is enhanced over 14.68 times than other molecules in flavonoids’ families (**Supplementary Table 6**). Analogously, flavonols meet rule 23, which contains pentose in position 1 and hydroxyl in position 3, 11 will increase the bioactivity for 32.37 times than others for the function of DNA topological change. Natural products such as kaempferol glycoside could targeting the DNA topoisomerase, which closely related with DNA replication and cell cycle (Vega et al., 2007; Baikar and Malpathak, 2010). Rule 24 indicate multiple oxygen methyl in side chain will benefit to the function of sphingolipid translocation. Moreover, rule 25 and rule 26 illustrate the different side chain components may have potential affects for regulation of prostaglandin biosynthetic process and vasoconstriction.

## Discussion

In this article, comprehensive analysis was proposed to explore the MOA of natural flavonoid products and results indicated that flavonoids could affect essential pathways in several categories such metabolism, genetic information processing, environmental information processing, cellular processes, organismal systems, and human diseases related pathways. Among them, the enrichment in human diseases-related pathways illustrated the multifaceted therapeutic applications of flavonoids which could affect multiple human diseases such as cancers, neuro-disease, diabetes and infectious diseases. By compared with other natural plant products, flavonoids could significantly enrich in the pathways of breast cancer, Huntington’s disease, Alzheimer’s disease, insulin resistance and drug resistance. Also, after systemically analysis of targets for different flavonoids subclasses, it can be found that targets such as MAPT, APEX1, and ALDH1A1, which were closely related with nervous system and cancer, were significantly enriched in almost all flavonoid subclasses. In that case, the multifaceted therapeutic ability indicates the utility of flavonoids for cancer and nervous system related drug discoveries.

Besides common biological functions, specific functions of different flavonoids subclasses were also analyzed and detected in both target and pathway level. For example, flavones and isoflavones were significantly enriched in multi-cancer related pathways than others, which indicate the potential therapeutic utility in cancer treatment. Also, flavan-3-ols were found on cellular processing and lymphocyte regulation, flavones specifically acted on cardiovascular related activities and isoflavones were closely related with cell multisystem disorders. Different structural and physic-chemical properties of natural flavonoid products may relate with the functional differences and can be detected in physic-chemical properties including H-bond acceptor, H-bond donor, lipid-water partition coefficient and topological polarity surface area. It can be noted that LogP and TPSA are closely related with absorption and distribution of chemical components in drugs, since appropriate solubility and lipid water distribution coefficient play essential roles in drug efficacy (Avdeef, 2001). For example, drugs which were activated in central nervous system requires larger liposolubility, which could be increased by non-polar structural fragments such as alkyl, halogen atom and aliphatic ring in chemical molecules. Meanwhile, TPSA can affect the cell penetration of drug molecules. Previous research indicated that in order to pass through the BBB and activate on the receptors in central nervous system, the polar surface areas of drug should be less than 90 square angstroms (van de Waterbeemd et al., 1998). Thus, natural products in flavonoids, flavones, flavanones, and isoflavones, which contains larger LogP and lower TPSA, have the ability to pass through the BBBs with potential activities.

Since flavonoids contain the same core scaffold, the functional difference was mainly related with the substituent groups. Relationship between chemical constitution fragment and biological effects indicated that different side chain can significantly affect the activity of flavonoids on the same target. Flavonoids with structures meet the corresponding rules will enhance the bioactivity of molecules for dozens of times. For 26 rules summarized in this article, the bioactivities were increased over three times at least. Among them, seven rules could enhance the bioactivities for over 10 times, and two rules (rule 23 and 26) could increase the activities for 30 times (**Supplementary Table 6**). Considering the substituent groups and positions of side chain, the relationship between structure and bioactivity analyzed in here may help to enhance the understanding of flavonoids and its potential ability for new drug discovery.

## Author Contributions

TQ and KT wrote the manuscript. DW and LY conceived and designed the experiments. HY and TQ analyzed and interpreted the results. TQ and QW modified the manuscript. ZC and KT supervised the project. All authors have read and approved the final version.

## Conflict of Interest Statement

The authors declare that the research was conducted in the absence of any commercial or financial relationships that could be construed as a potential conflict of interest.
