# Supplementary material for: Exploring the Mechanism of Flavonoids Through Systematic Bioinformatics Analysis
Source: Front Pharmacol. 2018 Aug 15;9:918. doi: 10.3389/fphar.2018.00918 (PMC6104453; doi:10.3389/fphar.2018.00918)
Supplement: Supplementary file 7 [file Image_1.pdf]

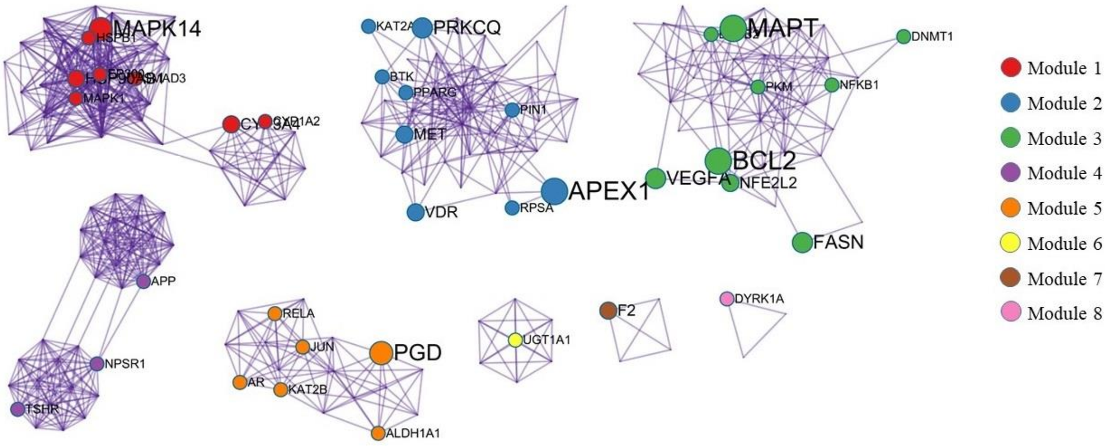

Supplementary Figure 1. Modularized PPI network of natural Flavan-3-ols products' targets. Size of each node represents the ratio (=number of target related compounds/total number of compounds) of targets. Different modules were marked in different colors.

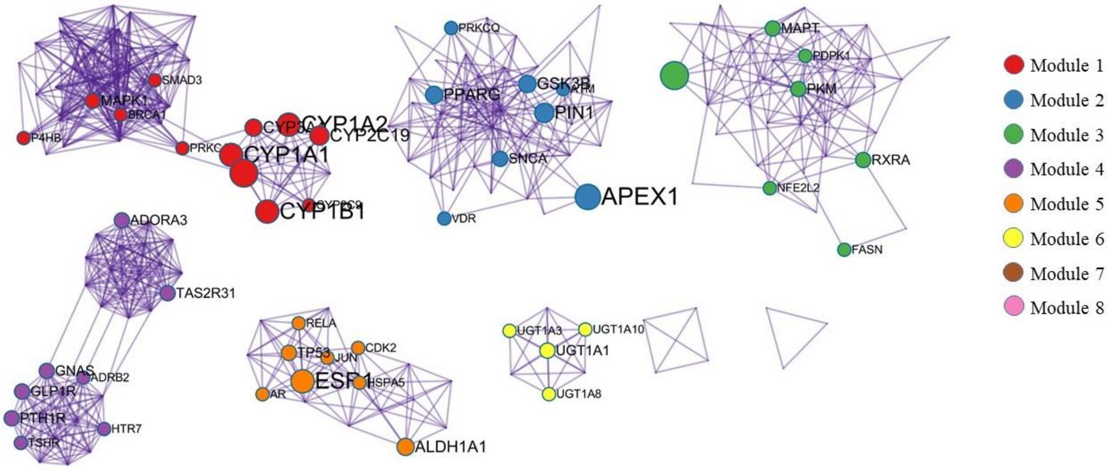

Supplementary Figure 2. Modularized PPI network of natural Flavanones products' targets. Size of each node represents the ratio (=number of target related compounds/total number of compounds) of targets. Different modules were marked in different colors.

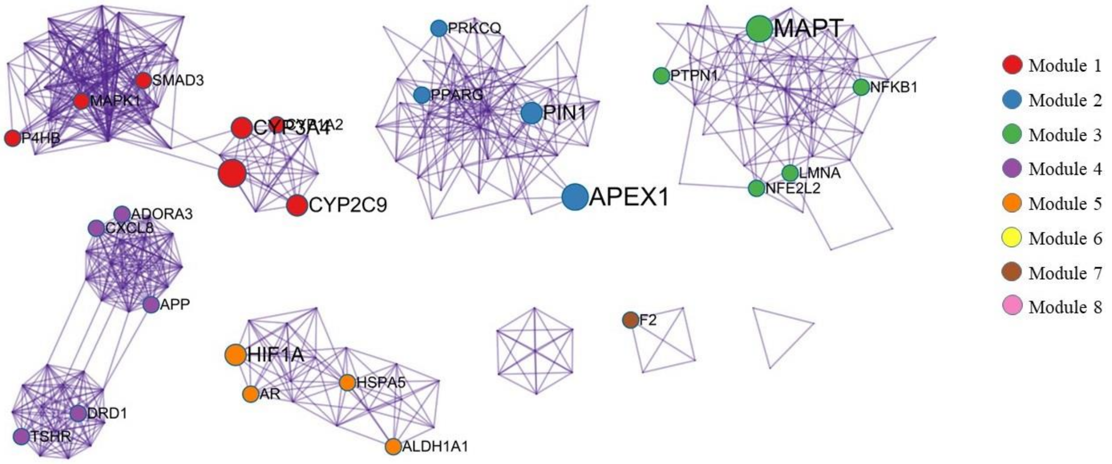

Supplementary Figure 3. Modularized PPI network of natural Flavanonol products' targets. Size of each node represents the ratio (=number of target related compounds/total number of compounds)

of targets. Different modules were marked in different colors.

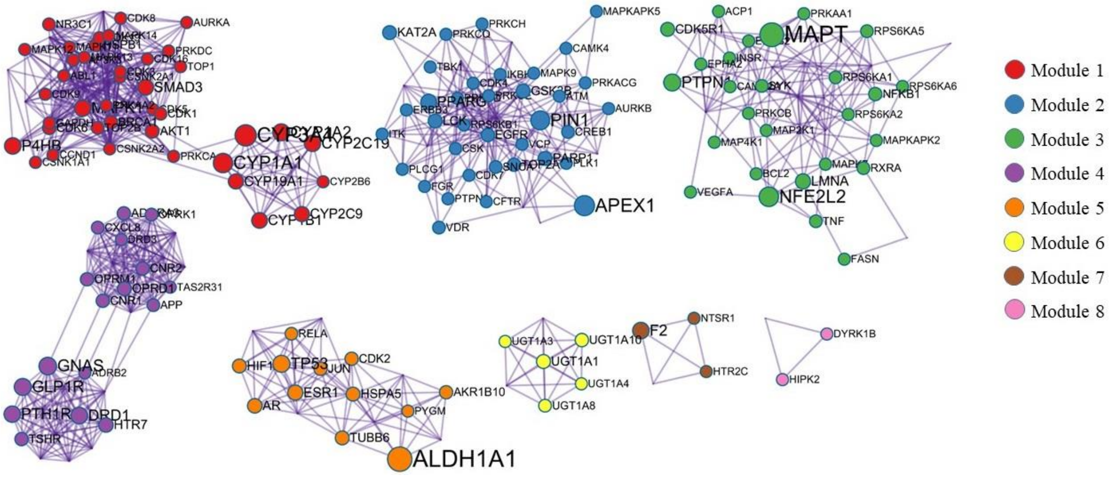

Supplementary Figure 4. Modularized PPI network of natural Flavones products' targets. Size of each node represents the ratio (=number of target related compounds/total number of compounds) of targets. Different modules were marked in different colors.

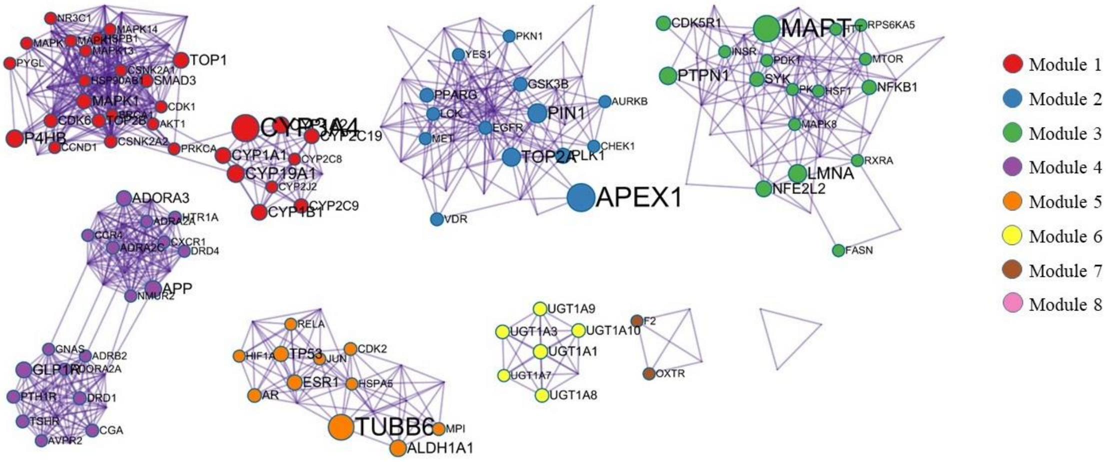

Supplementary Figure 5. Modularized PPI network of natural Flavonols products' targets. Size of each node represents the ratio (=number of target related compounds/total number of compounds) of targets. Different modules were marked in different colors.

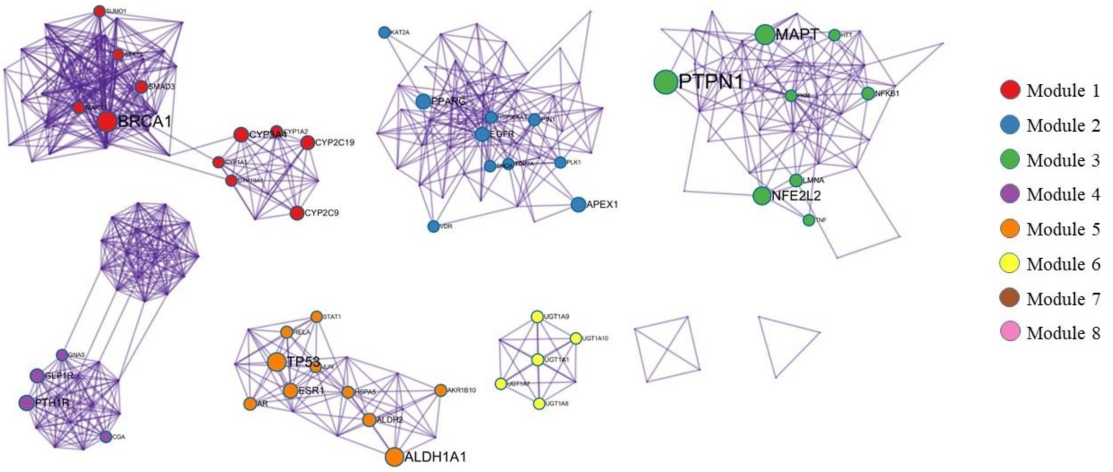

Supplementary Figure 6. Modularized PPI network of natural Isoflavones products' targets. Size of each node represents the ratio (=number of target related compounds/total number of compounds) of targets. Different modules were marked in different colors.

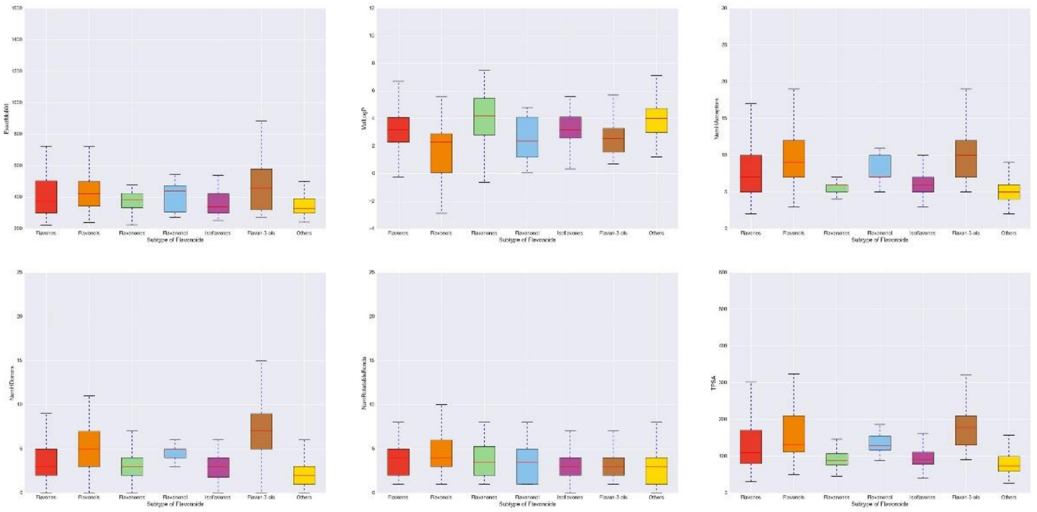

Supplementary Figure 7. Structure activity relationship analysis through 6 physic-chemical properties

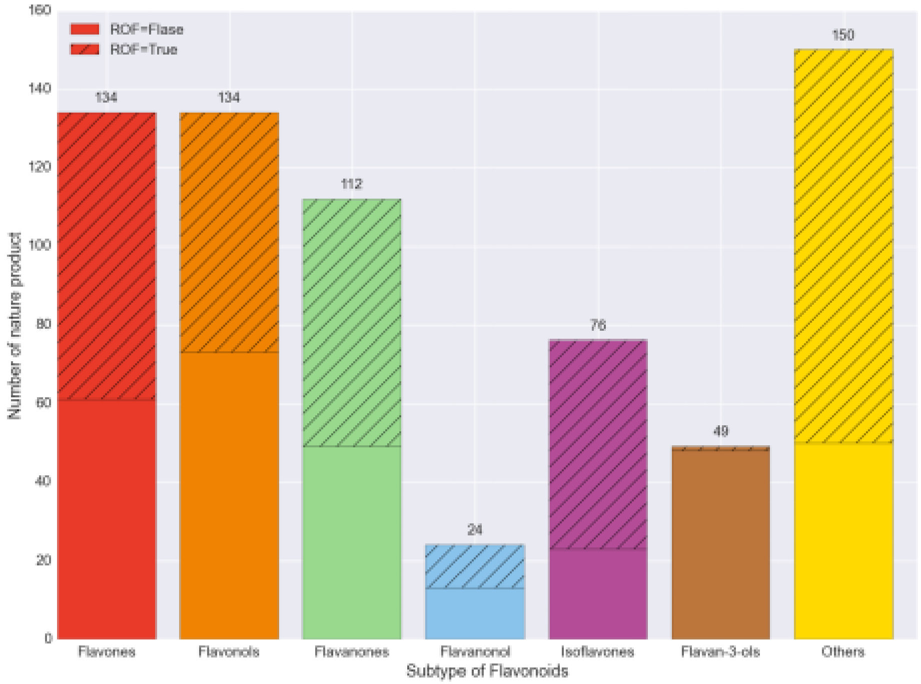

Supplementary Figure 8. Drug-likeness of different flavonoid subclasses based on ROF.
